# Supplementary material for: Causal Relationship between Aging and Anorexia Nervosa: A White-Matter-Microstructure-Mediated Mendelian Randomization Analysis
Source: Biomedicines. 2024 Aug 16;12(8):1874. doi: 10.3390/biomedicines12081874 (PMC11351342; doi:10.3390/biomedicines12081874)
Supplement: Supplementary file 1 [file biomedicines-12-01874-s001.zip › Supplementary Figure/Figure S8/Leaveoneout plot of MR analysis for TL to ubm-b-1522.pdf]

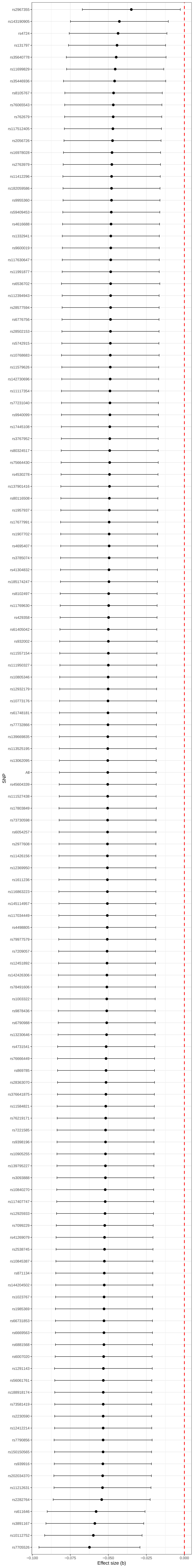
rs2967355
rs143190905
rs4724
rs131797
rs35640778
rs11699829
rs35446936
rs8105767
rs76065543
rs762679
rs117512405
rs2056726
rs16978028
rs2763979
rs11412296
rs182059586
rs9955360
rs59409453
rs4616688
rs1332941
rs9600019
rs117630647
rs11991877
rs6536702
rs112394943
rs28577594
rs6776756
rs28502153
rs5742915
rs10768683
rs11579626
rs142730696
rs11117354
rs77231040
rs9940099
rs17445108
rs3767952
rs80324517
rs75664430
rs4530278
rs137901416
rs80116508
rs1957937
rs17677991
rs1907702
rs4695407
rs3785074
rs41304832
rs185174247
rs8102497
rs11769630
rs429358
rs61405042
rs932002
rs11557154
rs111950327
rs10805346
rs12932179
rs10773176
rs61748181
rs77732866
rs139669835
rs113525195
rs13062095
A8
rs45604339
rs111527438
rs17803849
rs73730598
rs6054257
rs2977608
rs11426156
rs12369950
rs1611236
rs116863223
rs145114957
rs117034449
rs4498805
rs79977579
rs7209057
rs12451892
rs142426306
rs78491606
rs1003322
rs9878436
rs6790988
rs13230646
rs4731541
rs76666449
rs869785
rs28363070
rs376641875
rs11584821
rs76219171
rs7221585
rs9398196
rs10905255
rs139795227
rs3093888
rs10840270
rs117407747
rs12925933
rs7099229
rs41269079
rs2538745
rs10845387
rs871134
rs144204502
rs1023767
rs1985369
rs66731853
rs6669563
rs6881568
rs6007020
rs1291143
rs56061761
rs188918174
rs73581419
rs2230590
rs12412214
rs7790856
rs150150565
rs939916
rs202034370
rs11212631
rs2282764
rs611646
rs3891167
rs10112752
rs7705526
SNP
-0.100
-0.075
-0.050
-0.025
0.000
Effect size (b)
